# Perceptions, Attitudes, and Barriers to Human Papillomavirus Vaccination Among Residents in Saudi Arabia: A Cross-Sectional Study

**DOI:** 10.7759/cureus.57646

**Published:** 2024-04-05

**Authors:** Hind Faqeeh, Rahaf Alsulayyim, Kholoud Assiri, Mada Alqhatani, Asmaa Zanquti, Walaa Hakami, Khadijah Amri, Salva Akaram

**Affiliations:** 1 Obstetrics and Gynecology, AlKhamis Maternity and Children Hospital, Khamis Mushait, SAU; 2 Obstetrics and Gynecology, King Abdulaziz Hospital, Jeddah, SAU; 3 Obstetrics and Gynecology, Maternity and Children Hospital, Najran, SAU

**Keywords:** hpv, preventive behavior, saudi arabia, human papilloma virus vaccine, vaccination, cervical cancer

## Abstract

Background: Human papillomavirus (HPV) infection is a prevalent sexually transmitted infection globally, with significant associations with various cancers. Despite the availability of HPV vaccines, vaccination uptake remains suboptimal in many regions, including Saudi Arabia.

Methods: This cross-sectional survey-based study aimed to assess perceptions, attitudes, and barriers related to HPV vaccination among residents in different regions of Saudi Arabia. Participants were recruited using a stratified sampling method, and data were collected through a structured questionnaire administered electronically.

Results: A total of 600 participants were included in the study, with diverse demographic profiles observed. The largest age group was 26-35 years old, comprising 42.7% (n = 256) of the sample. The distribution of gender was approximately equal, with 54.7% (n = 328) male participants. Notable perception and knowledge about HPV were observed, with 49.7% (n = 298) reporting being very familiar. Furthermore, 85.8% (n = 515) believed that HPV could cause serious health problems, and 63.7% (n = 382) perceived the vaccine to be very effective. Positive attitudes toward HPV vaccination were prevalent, with a significant proportion expressing a willingness to recommend the vaccine to themselves (70.0%, n = 420) and their children (65.0%, n = 390). Healthcare provider recommendation emerged as a key influencing factor, with 73.8% (n = 443) citing it as influential. Despite favorable attitudes, barriers to HPV vaccination were reported, including vaccine cost (22.5%, n = 135) and lack of awareness (13.7%, n = 82). However, a substantial proportion of unvaccinated participants cited not applicable reasons (57.2%, n = 343), indicating prior vaccination. Factors associated with HPV vaccination uptake included higher education levels, with those holding a master's degree or above exhibiting higher odds of vaccination uptake (OR (odds ratio): 1.80, 95% CI (confidence interval): 1.35-2.40, p < 0.001). Age (26-35 vs. 18-25) also showed a borderline significant association (OR: 1.25, 95% CI: 0.98-1.60, p = 0.074).

Conclusion: The study highlights the importance of understanding perceptions and barriers to HPV vaccination to inform public health policies and programs in Saudi Arabia. Addressing identified barriers, such as educational campaigns and improving access to vaccination services, is essential for enhancing HPV vaccination coverage and reducing the burden of HPV-related diseases in the region.

## Introduction

Human papillomavirus (HPV) infection is one of the most common sexually transmitted infections globally, with a high prevalence among sexually active individuals. HPV infection is a significant public health concern due to its association with various cancers, including cervical, anal, vaginal, vulvar, penile, and oropharyngeal cancers [1]. Cervical cancer, in particular, is a leading cause of cancer-related morbidity and mortality among women worldwide, with an estimated 570,000 new cases and 311,000 deaths annually, the majority of which occur in low- and middle-income countries [2].

The development of HPV vaccines represents a significant breakthrough in cervical cancer prevention and control efforts [3]. HPV vaccines, primarily targeting HPV types 16 and 18, which are responsible for approximately 70% of cervical cancer cases, have demonstrated high efficacy in preventing HPV infection and associated precancerous lesions [4]. Vaccination programs targeting adolescent girls have been implemented in many countries, resulting in substantial reductions in HPV infection rates and related diseases [5-7]. However, despite the proven efficacy and safety of HPV vaccines, vaccine uptake remains suboptimal in many regions, including Saudi Arabia.

In Saudi Arabia, cervical cancer is the third most common cancer among women, with an estimated incidence rate of 5.2 per 100,000 women [8]. Despite the availability of HPV vaccines and the inclusion of HPV vaccination in the national immunization program since 2018, vaccination coverage remains low [9]. Several factors contribute to the low uptake of HPV vaccination in Saudi Arabia, including limited awareness and knowledge about HPV and cervical cancer, cultural and religious beliefs, concerns about vaccine safety and efficacy, and healthcare system-related barriers [10-13].

Understanding the perceptions, attitudes, and barriers related to HPV vaccination among residents in Saudi Arabia is crucial for developing targeted interventions to improve vaccine uptake and reduce the burden of HPV-related diseases. Therefore, this study aims to assess the knowledge, attitudes, and practices of HPV vaccination among residents in different regions of Saudi Arabia.

## Materials and methods

Study design

This research employed a cross-sectional survey-based approach to investigate perceptions and barriers related to HPV vaccination among residents in Saudi Arabia.

Study population

Participants were recruited using a stratified sampling method aimed at ensuring representation from diverse regions across Saudi Arabia, including the Eastern, Southern, Northern, Western, and Central Provinces. Recruitment efforts were conducted through a combination of online platforms and in-person recruitment at healthcare facilities, community centers, and educational institutions.

Data collection

Data were collected using a structured questionnaire designed to capture information on demographics, perceptions of HPV and HPV vaccination, knowledge about vaccination guidelines, attitudes toward vaccination, and barriers to vaccination. The questionnaire consisted of items developed based on a comprehensive review of existing literature on HPV vaccination. It underwent several rounds of piloting and refinement to ensure clarity, relevance, and comprehensibility. The questionnaire was administered electronically through online survey platforms. Participants were assured of the confidentiality and anonymity of their responses, and informed consent was obtained from all participants before they participated in the study.

Data analysis

Descriptive statistics, including frequencies and percentages, were used to summarize demographic characteristics, perceptions, attitudes, and barriers related to HPV vaccination. Additionally, a multivariable logistic regression analysis was conducted to identify factors associated with HPV vaccination uptake. Odds ratios (ORs) and 95% confidence intervals (CIs) were calculated to assess the strength and direction of associations between independent variables and vaccination uptake.

## Results

Demographic characteristics

A total of 600 participants were included in the study, with diverse demographic profiles (Table 1). The largest age group was 26-35 years old, comprising 42.7% (n = 256) of the sample. The gender distribution was nearly balanced, with 54.7% (n = 328) male participants. Regarding education level, 47.2% (n = 283) held a bachelor's degree, while 43.2% (n = 259) were healthcare professionals. The majority of participants resided in the Western Province (28.0%, n = 168).

**Table 1:** Demographic characteristics of participants (N=600) The table provides information regarding the demographic characteristics of 600 participants. Each category is reported in terms of both frequency (n) and percentage (%). Percentages may not sum to 100% due to rounding.

Variable		Frequency	Percentage
Age	18-25	183	30.5
	26-35	256	42.7
	36-45	121	20.2
	46-55	35	5.8
	56 and above	5	0.8
Gender	Male	328	54.7
	Female	272	45.3
Education level	High school or below	148	24.7
	Bachelor's degree	283	47.2
	Master's degree or above	169	28.2
Occupation	Student	178	29.7
	Healthcare professional	259	43.2
	Non-healthcare professional	163	27.2
Region	Eastern province	117	19.5
	Southern province	153	25.5
	Northern province	102	17.0
	Western province	168	28.0
	Central province	60	10.0

Perception and knowledge about HPV and HPV vaccination

Participants demonstrated a high level of familiarity with HPV, with 49.7% (n = 298) reporting being very familiar (Table 2). Furthermore, 85.8% (n = 515) believed that HPV could cause serious health problems. Regarding HPV vaccination, 63.7% (n = 382) perceived the vaccine to be very effective. Knowledge about vaccination recommendations was notable, with 57.5% (n = 345) identifying two doses as the recommended regimen.

**Table 2:** Perception and knowledge about HPV and HPV vaccination The table provides insights into perceptions and knowledge about human papillomavirus (HPV) and HPV vaccination. Responses are presented alongside their frequencies and percentages. Please note that, due to rounding, percentages may not sum to 100%.

Survey question			Response	Percentage
Familiarity with HPV		Very familiar	298	49.7
		Somewhat familiar	242	40.3
		Not familiar	60	10.0
Belief in HPV causing serious health problems		Yes	515	85.8
		No	48	8.0
		Unsure	37	6.2
Opinion on HPV vaccine effectiveness		Very effective	382	63.7
		Somewhat effective	195	32.5
		Not effective	23	3.8
Knowledge about HPV vaccination	Recommended doses for complete protection	One dose	63	10.5
		Two doses	345	57.5
		Three doses	192	32.0
	Ideal vaccination age range	Before 9 years old	85	14.2
		9-14 years old	422	70.3
		15-26 years old	87	14.5
		Any age	6	1.0

Attitudes toward HPV vaccination

Participants exhibited positive attitudes toward HPV vaccination. A significant proportion expressed willingness to recommend the vaccine to themselves (70.0%, n = 420) and their children (65.0%, n = 390). Healthcare provider recommendation emerged as a key influencing factor, with 73.8% (n = 443) citing it as influential in their decision-making process. Despite favorable attitudes, participants encountered barriers to HPV vaccination. The cost of the vaccine (22.5%, n = 135) and lack of awareness (13.7%, n = 82) were notable obstacles. However, a substantial proportion of unvaccinated participants cited not applicable reasons (57.2%, n = 343), indicating prior vaccination (Table 3).

**Table 3:** Attitudes toward HPV vaccination The table provides insights into attitudes toward human papillomavirus (HPV) vaccination. Responses to survey questions are presented alongside their frequencies and percentages. Please note that, due to rounding, percentages may not sum to 100%.

Survey question		Response	Percentage
Would you recommend the HPV vaccine to	Yourself	420	70.0
	Your children (if applicable)	390	65.0
	Other family members or friends	312	52.0
	None of the above	30	5.0
	I'm not sure	48	8.0
Encountered barriers to HPV vaccination	Lack of access to healthcare facilities	80	13.3
	Cost of the vaccine	135	22.5
	Fear of needles or injections	92	15.3
	Concerns about vaccine safety	110	18.3
	Lack of awareness about HPV and the vaccine	82	13.7
	Cultural or religious beliefs	58	9.7
	Other	43	7.2
	None	10	1.7
Reasons for not being vaccinated against HPV	Lack of access to healthcare facilities	35	5.8
	Cost of the vaccine	82	13.7
	Fear of needles or injections	48	8.0
	Concerns about vaccine safety	62	10.3
	Lack of awareness about HPV and the vaccine	65	10.8
	Cultural or religious beliefs	42	7.0
	Other	23	3.8
	Not applicable (I have been vaccinated)	343	57.2

Factors associated with HPV vaccination uptake

The multivariable logistic regression analysis identified factors associated with HPV vaccination uptake (Table 4). Participants with a master's degree or above exhibited higher odds of vaccination uptake (OR: 1.80, 95% CI: 1.35-2.40, p < 0.001), while age (26-35 vs. 18-25) showed a borderline significant association (OR: 1.25, 95% CI: 0.98-1.60, p = 0.074).

**Table 4:** Multivariable logistic regression analysis of factors associated with HPV vaccination uptake The table presents results from a multivariable logistic regression analysis investigating factors associated with human papillomavirus (HPV) vaccination uptake. Odds ratios with 95% confidence intervals (CI) and corresponding p-values are reported for each variable. An asterisk (*) denotes a significant p-value < 0.05, indicating statistical significance.

Variables	Odds ratio (95% CI)	P-value
Gender (female vs. male)	1.12 (0.85-1.48)	0.408
Age (26-35 vs. 18-25)	1.25 (0.98-1.60)	0.074
Education level (master's or above vs. high school or below)	1.80 (1.35-2.40)	<0.001*
Occupation (healthcare professional vs. student)	0.98 (0.75-1.28)	0.886
Region (western province vs. eastern province)	1.05 (0.79-1.40)	0.729

## Discussion

The findings of this study provide valuable insights into the perceptions, attitudes, and barriers related to HPV vaccination among residents in Saudi Arabia. The study revealed a high level of awareness and knowledge about HPV and HPV vaccination among participants, with a majority recognizing HPV as a significant cause of cervical cancer and perceiving the vaccine to be effective. These findings are consistent with previous research indicating increasing awareness of HPV and its association with cervical cancer globally [3-7]. The positive perception of HPV vaccination underscores the importance of continued efforts to educate the public about the benefits of vaccination in preventing HPV-related diseases [14].

Participants exhibited generally positive attitudes toward HPV vaccination, with a willingness to recommend the vaccine to themselves, their children, and others. Healthcare provider recommendations emerged as a significant influencing factor, highlighting the crucial role of healthcare professionals in promoting vaccine uptake. These findings align with previous studies emphasizing the influence of healthcare provider recommendations on vaccination decisions and underscore the importance of healthcare provider education and advocacy efforts [15].

Despite favorable attitudes, participants reported various barriers to HPV vaccination, including concerns about vaccine safety, a lack of awareness, and logistical challenges such as cost and access to healthcare facilities. These barriers are consistent with those identified in previous studies conducted in diverse settings and underscore the multifaceted nature of challenges to vaccine uptake. Addressing these barriers will require targeted interventions, including educational campaigns to dispel misconceptions about vaccine safety and efficacy, improving access to vaccination services, and addressing financial barriers through subsidized or free vaccine programs [5,8].

The multivariable logistic regression analysis identified several factors associated with HPV vaccination uptake, including education level. Participants with higher education levels exhibited higher odds of vaccination uptake, highlighting the importance of education in shaping health behaviors and decision-making. Age also showed a borderline significant association with vaccination uptake, suggesting potential differences in vaccination behavior across age groups. These findings underscore the need for targeted interventions tailored to specific demographic groups to optimize vaccine uptake.

Strengths and limitations

This study has several strengths, including its comprehensive assessment of perceptions, attitudes, and barriers related to HPV vaccination among residents in Saudi Arabia. However, it is not without limitations. The cross-sectional study design precludes establishing causality, and the reliance on self-reported data may introduce response bias. Additionally, the use of convenience sampling may limit the generalizability of the findings to a broader population.

## Conclusions

This study sheds light on the perceptions, attitudes, and barriers surrounding HPV vaccination among residents in Saudi Arabia. Despite a generally positive perception of HPV vaccination and high levels of awareness about its role in preventing cervical cancer, several barriers hinder optimal vaccine uptake, including concerns about safety, lack of awareness, and logistical challenges. Healthcare provider recommendations emerged as a critical factor influencing vaccination decisions, highlighting the importance of provider education and advocacy efforts. Targeted interventions addressing identified barriers, such as educational campaigns and improving access to vaccination services, are essential for enhancing HPV vaccination coverage and reducing the burden of HPV-related diseases in Saudi Arabia. Collaborative efforts involving healthcare professionals, policymakers, and community stakeholders will be crucial in advancing HPV vaccination efforts and promoting public health in the region.
